# On the Entozoaria, Found in the Eye of Some Mammalia, and of Man in Particular

**Published:** 1841-06-26

**Authors:** J. Trudeau


### MEDICAL EXAMINER.

DEVOTED TO MEDICINE, SURGERY, AND THE COLLATERAL SCIENCES.

No. 26.] PHILADELPHIA, SATURDAY, JUNE 26, 1841. [Vol. IV.

### ORIGINAL COMMUNICATIONS.

### *On the Entozoaria found in the eye of some Mammalia, and of Man in particular.* By J. Trudeau, M. D.

In the organs of most animals, and in those of the vertebrated class in particular, other animals of an inferior order are found. They are divided in two great classes, termed *epizoaria* when found superficially on the surface of the body, and *entozoaria* when they inhabit the deep-seated organs, as the brain, liver, uterus, intestinal canal, &c. A few years ago, the publication of the works of Rudolphi and of Bremser threw great light on their history, so little known at that time. To Von Ammon, to his pupil Gescheidt, and especially to the celebrated naturalist Nordman, we are indebted for the discovery of several new species found in the human eye, and also in the eyes of some other vertebrated animals. Indeed, so little is known to the profession on this subject, which has only attracted the attention of naturalists, that, in his able article in the Dict. de Médecine, (25 vol.) M. Guérard seems not to be aware of the existence of insects in the human eye. All the other treatises of special and general pathology, except Von Ammon's, seem to be no better informed. Dr. Gescheidt, of Dresden, has published an excellent paper on the entozoaria found in the eyes of quadrupeds, in the last number of Von Ammon's Journal. I will try to collect together all the observations published on this subject, with some curious facts lately observed by myself.

*Of the Entozoaria found in the eyes of animals.*—Gessner describes and delineates from Rhodius, (obs. 33, page 45, Ann. 1676,) a large worm found in the vitreous body of a horse's eye. It is as large as a crow-quill, and 1½ inches in length. Gessner terms it the "Vitulus Aquaticus Minimus." Schulze, (Ephem. Naturæ Curios., Ann. II. p. 43,) in a paper entitled "De Vermibus in Vivor. Crysorit," relates that a worm of the size of a bombyx was extracted from the eye of a peasant boy, by the surgeon Caspar Wendlandt. The patient was relieved, but lost his eye afterwards.

Mongin, (Sur un ver trouvé sous la conjonctive, Journ. de Méd. de Paris, tom. xxxii. Ann. 1770,) a French physician at St. Domingo, relates that he discovered a worm between the tunica albuginea and the conjunctiva, on a black woman. The pains suffered by the patient were excruciating, and had begun twenty-four hours previous. The eye, however, was not inflamed. The conjunctiva being open, the worm was easily extracted. It was one inch in length, as thick as a small violin string, of a grayish colour, and dotted to both extremities.

Bajon (Mém. pour Servir à l'hist. de la Guyane francaise, pp. 89 and 122, Ann. 1777,) relates also several cases which may be considered as the fac-simile of Mongin's case. But as it is self evident that the worms alluded to are the filaria medinensis, and not insects proper to the eye, I will not quote any of his notes.

The presence of insects in the eyes of quadrupeds is now well known to naturalists, and attested by many writers. Hopkins (Trans. of the Am. Philos. Soc., vol. ii., Ann. 1786,) is the first who gives a full and correct account of a worm found in the eye of a horse. Morgan (ibid. p. 386) records another case, similar to the fact above quoted from Rhodius. These two cases seemed to have attracted the attention of the European naturalists, and from that time numerous cases were recorded by Brown and Twining, in England; Chaignaud, Desmarests and Fermsac, in France; and, lately, Rudolphi, Gurlt and Von Ammon, in Germany, have related such facts as to leave no doubt respecting the truth of their assertions.

In the numerous cases recorded by these authors, (Chaignaud relates about 150 cases,) no mention is made whether the eye was diseased previous to the discovery of the worm. But they all agree in saying that the insect had done serious damage in the eye, as thickening of the lids, inflammation of the conjunctiva—in some cases, spots and ulcerations on the cornea. In many of these, the iris and crystalline were destroyed, the humours evacuated, and the sight lost. These accidents always happen when the filaria is not timely extracted.

Dr. Gescheidt mentions in his paper, that in India the filaria papillosa exists among horses and oxen under an epidemic form, and that the species found in the eye is always found in the lumbar region, sometimes in the spine, seldom in the brain.

Nordman [Microscop. researches, (in German) 1832,] found the following new species in the serea fluviatilis:—the oxyuris velocissima, deplostomum volvens and clavatum, and the holostomum brevicandatum.

The same author, in the above quoted work, asserts that the cysticercus cellulosa is frequently found in the eyes of pigs. Dr. Gescheidt, on eighteen diseased eyes of dogs, (cataract,) found a filaria, called by him F. oculi lamis.

The filaria is seldom met with in birds.

Nordman found a new species in the eye of the tetrao bonasia; Dr. Gescheidt accidentally discovered it in the eye of the faleo lagomus. Though I have for many years directed my attention towards this subject, and have examined more than three hundred eyes of different species of birds, I have never found any helmenithi. But I have seen on a mocking bird alive in the Academy of Natural Sciences of Philadelphia, a large filaria. It had almost entirely destroyed the eye of the bird, and seemed to be very active. As the bird was kept by the members so as to give them a chance of observing it, I have not been able to procure the worm. It is undoubtedly a new species, for which I propose the specific appellation of *filaria armata.*

In 1836, I shot in Jersey a common hare, (Lyrus Virginianus) whose eye was mostly destroyed by a filaria. The worm, four lines in length, was of pure white, and in size very much like a thread. It emerged from a wide ulceration of the cornea, and was dead when I killed the hare. This species I think is also new, and in case it should prove so, I would propose for it the name of *filaria Oculi Lynoris.*

I found in Louisiana last year, several small worms in the right eye of a ram which died of old age. They must be referred, I think, to the genus *ascaris.* They were eight in number in the vitreous body; the length was about half a line. These small insects seemed to be active and full of life, though the ram had been dead for more than two hours.

In the year 1840 I had frequent occasions of observing the entozoaria of the eyes of horses and of buffaloes in the prairies of the southwestern district. The filaria papillosa seems to be the most common. I have found it nine times on old buffalo bulls on a number of eyes, not less than 182. It never existed but in one eye. On that number, five eyes were affected with lenticular cataract; but I did not notice any disorganization of the eye. The largest of the F. papillosa found was more than one inch long, and about one-eighth of a line thick. All these insects were dead, and found in the vitreous body.

Among the Indian horses, the *filaria papillosa*, and another smaller species, which I have described under the name of *filaria lentis*, are not uncommon. The Indians are well informed of the fact, and have pointed out several cases to me.

All the members of the medical profession have had an opportunity of observing a large F. papillosa in the eye of a horse exhibited at the Tattersall's of this city, as having a living snake in its eye.

*On the Entozoaria found in the human eye.*—The presence of insects in the human eye must determine a number of diseases and of symptoms, sometimes of great severity, of which, so far, the cause was not known. Several of these parasitic beings cannot be detected without the assistance of the magnifying glass, and even of a powerful microscope. To Nordman and Gescheidt we are indeed indebted for having called the attention of the profession to the various disorders occasioned by this class of worms.

The first authentic case of entozoaria found in the human eye is the well-known fact of Sœmmering, (Tris, 1830, vol. vii. p. 717.) A girl of eighteen years of age had in the anterior chamber of the eye, a *cysticercus cellulosa* alive. That worm seemed to have been formed after a violent ophthalmia. The figure given by Sœmmering was taken when the animal could be detected with the naked eye, two months after the inflammation had subsided. This foreign body occasioned but a trifling pain while moving, and interfered with the sight only when in a line with the pupil. As it grew very large, and in consequence was troublesome to the patient, it was extracted by a small incision of the cornea.

Mr. M'Kenzie, of Edinburgh, has also lately observed a fact much like the one related above, on a little girl nine years old. The insect appeared six weeks after a violent inflammation of the eye, as a small globule floating in the aqueous fluid. It was extracted by the same means.

Rossi (Mem. dell Real Acad. del Sc. Torin, &c.) mentions that he had frequently discovered hydatids in the eye, between the choroid and retina.

Nordman (Microscopic research to compare the history of invertebrated animals, in German,) has detected the *filaria oculi humani* in the aqueous fluid of an aged man affected with a capsular cataract. This singular threadworm was three-fourths of an inch in length. The same author found another filaria in the crystalline of an old woman. It was about one inch long.

Dr. Gescheidt observed a small filaria in the lens crystallina of a man operated on for cataract by extraction, by Prof. Von Ammon.

The distoma oculi humani was observed and described by Dr. Gescheidt. Four or five of these small worms were found betwixt the lens crystalline and its capsule, on a child affected with congenital cataract, and who died of marasmus. Their size varied from one-fourth to one-half of a line in length, and their tenuity such as not to be measured.

Several other small microscopic worms were found in the choroid, and described by Nordman, as the echinnoccus hominis, diplostomum volvens, and monostoma lentis.

Before ending this paper, I propose examining, superficially, the question of the formation of entozoaria in the eye. It is needless to mention here, that the opinion of helminthologists is divided on this point. Rudolphi thinks that the eggs of the worms are carried by circulation and absorption into the remotest parts of the body. It seems on that hypothesis difficult to explain how the worms are found in the anterior and posterior chambers. Bremser, Nordman, Gescheidt, don't admit the possibility of Rudolphi's opinion, and think that their presence is owing to spontaneous generation. In most all the cases above reported, we see that the appearance of the insect in the eye is preceded by some inflammation of the organ. No doubt that the diseased state of secretion and absorption, and consequently the inflammation, have had a great influence on the formation of the entozoaria. Are not the entozoaria products of inflammation?

The various disorders oscasioned by the presence of entozoaria in an organ of such a delicate and complicated structure as the eye, have been exposed at a proper place, and are easily understood. Inflammation of the eye, ulceration of the cornea, and, finally, loss of sight, are common consequences of their presence. The filarious medinensis and echinoccus, have been the causes of severe oppthalmia. In the horses and oxen, the filaria papillosa have destroyed the organ. In the three cases of filaria found on birds, they have caused death. A timely extraction would, without any doubt, always prevent such formidable accidents.

From what I have exposed, it is hardly necessary to mention, that the therapeutic indications are to remove the foreign body, and to lessen the inflammation by appropriate means. Some authors, Chaignaud in particular, strongly recommends to inject in the eye a strong decoction of tobacco leaves. It might prove useful, but I cannot understand upon what ground; especially when the worm is deeply buried in the tunics, and hardly endowed with life, as it is the case with the genius *cysticercus* and the *acepolocysts*. Rudolphi extols highly the internal use of the muriate of barytes, and Gurlt the muriate of soda. They say that they have by these means killed the worm, which was subsequently removed by absorption. It may be true: and the remedy must certainly be tried in saying with Celsus—Melius anceps quam nullum remedium.

